# Hospital admissions in infants with Down syndrome: a record‐linked population‐based cohort study in Wales

**DOI:** 10.1111/jir.12903

**Published:** 2021-12-03

**Authors:** R. A. Esperanza, A. Evans, D. Tucker, S. Paranjothy, L. Hurt

**Affiliations:** ^1^ School of Medicine Cardiff University Cardiff UK; ^2^ Cwm Taf Morgannwg University Health Board Merthyr Tydfil UK; ^3^ Division of Population Medicine, School of Medicine Cardiff University Cardiff UK; ^4^ Congenital Anomaly Register and Information Service Public Health Wales Swansea UK; ^5^ Centre for Health Data Science University of Aberdeen Aberdeen UK

**Keywords:** Children, Co‐morbidity, Down syndrome, Hospitalisations, Infants

## Abstract

**Background:**

Despite recent advances, mortality in children with Down syndrome remains five times higher than in the general population. This study aims to describe the burden, patterns and causes of hospital admissions in infants with Down syndrome, and compare this with infants without Down syndrome in a population‐based cohort.

**Methods:**

This study used data from the Wales Electronic Cohort for Children, a cohort of all children born in Wales between 1990 and 2012. The cohort was generated from routine administrative data, linked to create an anonymised data set within the Secure Anonymised Information Linkage databank. This analysis is based on all infants born between January 2003 and January 2012 who were followed to their first birthday, a move out of Wales, death, or until 31 October 2012 (end of follow‐up). Infants with Down syndrome were identified using the Congenital Anomaly Register and Information Service in Wales. Multivariable Cox regression was used to compare the time to first hospital admission. Admission codes were used to identify the commonest indications for hospitalisation and to determine the presence of other congenital anomalies.

**Results:**

We included 324 060 children, 356 of whom had Down syndrome. Of infants with Down syndrome, 80.3% had at least one hospital inpatient admission during the first year of life, compared with 32.9% of infants without Down syndrome. These first admissions were earlier [median of 6 days interquartile range (IQR) (3, 72) compared with 45 days [IQR 6, 166)] and longer [median of 4 days (IQR 1, 15) compared with 1 day (IQR 0, 3)] than in infants without Down syndrome. The most common causes of admissions were congenital abnormalities, respiratory diseases, conditions originating in the perinatal period and infectious diseases. The presence of other congenital abnormalities increased hospitalisations in all infants, but more so in infants with Down syndrome who spent a median of 21 days in hospital (IQR 11, 47) during their first year of life.

**Conclusion:**

Infants with Down syndrome are at high risk for early, more frequent and longer hospital admissions. Congenital heart disease and respiratory infections remain a major burden in this population. More research is needed to understand how to better manage these conditions particularly in the first month of life when most admissions occur.

## Background

Down syndrome is caused by a chromosomal abnormality that occurs as a result of acquiring an additional full or partial copy of chromosome 21. Approximately 1 in 1000 babies are born with Down syndrome worldwide (Weijerman and Winter 2010). In the UK, the population prevalence is 2.7 per 1000 (Morris and Springett 2014). The characteristic profile of Down syndrome includes several dysmorphic features, developmental delay and associations with multiple morbidities. It is the most common genetic cause of intellectual disability, accounting for approximately 12.5% of cases (Bittles *et al*. 2002). Children with Down syndrome are at higher risk of low birthweight, pre‐term deliveries and cardiac defects, leading to complications and increased mortality (Goldman *et al*. 2011; Kucik *et al*. 2013; Glasson *et al*. 2016). The survival of children with Down syndrome has recently increased as a result of earlier detection of Down syndrome and, therefore, a quicker response in management and treating those with cardiac anomalies (Glasson *et al*. 2016). Over 90% of children now survive to 10 years (Glasson *et al*. 2002) and 88% survive to 20 years (Kucik *et al*. 2013). Life expectancy, although still shorter than in individuals without Down syndrome, has also increased, to an average of 59 in 2002 (Glasson *et al*. 2002), compared with 12 years in 1949 (Penrose 1949). Despite this, the overall fatality rate among children with Down syndrome remains high, at more than five times than the general population (Weijerman *et al*. 2008). To ensure that children with Down syndrome are receiving appropriate and timely health care, it is important to understand the causes of ill‐health from early life.

In addition to congenital heart disease, Down syndrome is known to be associated with several other conditions; gastrointestinal malformations, vision and hearing problems, hypothyroidism and haematological disorders (Bull 2011; Kinnear *et al*. 2018). Congenital cardiac defects are present in approximately 50% of newborns with Down syndrome (Freeman *et al*. 2008; Irving and Chaudhari 2012) and remain a strong predictor of mortality (Frid *et al*. 1999; Yang *et al*. 2002). Furthermore, data suggest that respiratory tract infections are common in children with Down syndrome and a major cause of mortality (Yang *et al*. 2002; Bloemers *et al*. 2007). There is evidence that individuals with Down syndrome have multiple immunological abnormalities, such as reduced T‐cells and premature diminution of the thymus (Bloemers *et al*. 2010), which may in part explain their increased susceptibility to infections and autoimmune diseases.

Previous studies have investigated trends in hospital admissions in children with Down syndrome. These have suggested that the majority (79%) began their hospital journey before the age of 1 year (Fitzgerald *et al*. 2013). The frequency of hospital admissions reduced with age (So *et al*. 2007; Fitzgerald *et al*. 2013; Zhu *et al*. 2013; Dawson *et al*. 2014) and there is a higher risk of readmission in children with Down syndrome (Hughes‐McCormack *et al*. 2020). However, the majority of these studies were retrospective case series without a reference cohort of children without Down syndrome from the same population. There are limited data from the UK regarding hospital admissions in children with Down syndrome. There is also currently little evidence on the healthcare burden from having co‐occurring morbidities. More research in this area is necessary to understand why the mortality rate remains high in this population despite post‐natal screening and early management of common conditions that develop in early childhood (Bull 2011; Down Syndrome Medical Interest Group 2018). This information can be used to implement better supportive care pathways for children with Down syndrome, ensuring that they meet their needs and those of their families.

The aim of this study was to describe the burden, patterns and causes of hospital admissions (first, total and by cause) in children with Down syndrome in the first year of life, compared with children without Down syndrome. A secondary aim was to explore the influence of other co‐occurring congenital anomalies on hospitalisation patterns.

## Methods

### Study design and population

This cohort study used data from the Wales Electronic Cohort for Children (WECC), which includes all children in Wales or with a mother who is a resident in Wales.

### Data sources

The WECC was generated from routine administrative data, linked to create an anonymised data set within the Secure Anonymised Information Linkage (SAIL) databank (Ford *et al*. 2009). The data sets used to create the cohort are summarised in Table 1; additional details on data sets relevant to this analysis are given below. The cohort includes 981 404 children born between January 1990 and October 2012 to a mother normally resident in Wales. This was created to enable and further develop research using routinely collected data in Wales to inform child population health policy (Hyatt *et al*. 2011). Within each data set, an Anonymised Linking Field, based on encrypted National Health Service (NHS) numbers provided by NHS Wales Informatics Service, is assigned to each individual allowing for record‐linking of the data (Lyons *et al*. 2009). Children's records are linked using a deterministic record linkage system based on NHS numbers; however, if these are missing or incomplete, probabilistic matching based on names is employed. The SAIL linkage system is more than 99.85% accurate (Lyons *et al*. 2009). Approval for this analysis was obtained from the Information Governance Review Panel at SAIL. The Research Ethics Committee for Wales judged the Wales Electronic Cohort for Children to be an anonymised research database that does not require ethical review, in line with National Ethics Committee guidance.

**TABLE 1:** Data sources for WECC used in this analysis

Source	Information
Welsh Demographic Survey (from 1960) (formerly National Health Service Administrative Register)	All Wales population register that provides demographic characteristics of people registered with a General Medical Practitioner in Wales.
National Community Child Health Database (from 1987)	A national database of all children resident or born in Wales, containing information on birth characteristics such as gender, birth weight and mode of delivery.
Office of National Statistics Births Registry (from 2003)	Data on all births in Wales or children who are born to mothers usually resident in Wales.
Office of National Statistics Deaths Registry (from 2003)	Data on all deaths in Wales or of usual inhabitants in Wales.
All‐Wales Perinatal Survey (from 1993)	A database that holds information on infants from 20 weeks gestation to 1 year of age who die in a Welsh hospital or whose mother is usually resident in Wales.
Congenital Anomaly Register and Information Service (from 1998)	A population‐based register containing information on any foetus or baby with a congenital anomaly and whose mother was normally resident in Wales at the time of birth.
Patient Episode Database for Wales (from 1998)	A database containing all inpatient and day‐case admissions in National Health Service hospitals in Wales and further information on treatment received by Welsh residents in other UK countries.

### Participants and variables

#### Inclusion and exclusion criteria

This analysis is based on all infants born between January 2003 and January 2012 who were followed to their first birthday, a move out of Wales, death, or until 31 October 2012 (end of follow‐up). The time period for this study was selected due to availability of data on stillbirths (available from 2003) to ensure that these could be excluded from the analysis. Infants not born in Wales but who subsequently moved into Wales were also excluded due to high levels of missing data on all variables of interest.

#### Identification of infants with Down syndrome

Cases of Down syndrome were identified using the Congenital Anomaly Register and Information Service (CARIS) for Wales database, which collects information on all children with a confirmed or suspected congenital anomaly born to mothers who are normally resident in Wales (Table 1). A multiple source system is used in the register to maximise case finding. The most important sources of information are reports from healthcare workers, as these provide additional detail on the anomaly. Other sources include data from screening or diagnostic databases such as cytogenetics, radiology and specialist services such as paediatric cardiology (Congenital Anomaly Register and Information Service 2020). Information from multiple sources are triangulated by CARIS, and discrepancies are resolved by review. All cases of Down syndrome registered on this database in infants in the cohort were included in this analysis.

#### Definition of outcome

All inpatient hospital admissions for any cause in the first year of life were included in the analysis. An admission is defined as any length of continuous stay using a hospital bed provided by the NHS in Wales under one or more consultants, including transfers between hospitals as long as these transfers occur less than 1 day apart. Data on hospitalisations were obtained by data linkage to the Patient Episode Database for Wales (PEDW, Table 1).

#### Classification of admission

Hospital admissions were classified as elective, emergency or other admissions, and by cause. Admissions classified as ‘other’ include a transfer of any admitted patient from another hospital provider, or the birth of a baby outside of the healthcare provider with the admission required for observation, and therefore not considered an emergency (National Health Service Wales Informatics Service 2020). The latter category can also include infants born by caesarean section, where the initial admission is considered to be for the mother (who is post‐operative), but the baby develops a complication whilst in hospital after birth, and is considered as a new ‘admission’ within PEDW.

#### Cause of admission

Up to 14 diagnostic codes, based on the 10th revision of International Classification of Diseases (ICD‐10), are recorded in PEDW for each consultant episode of an admission. We used the first non‐R (‘symptoms, signs and abnormal clinical and laboratory findings, not elsewhere classified’) or Z (‘factors influencing health status and contact with health services’) diagnosis code in the first consultant episode of each admission as the primary reason for admission. These were first grouped into disease categories using the ICD‐10 chapter headings, and where there were enough admissions per group, specific ICD‐10 codes within the chapter were also examined.

#### Potential confounders, covariates and effect modifiers

**FIGURE 1:**
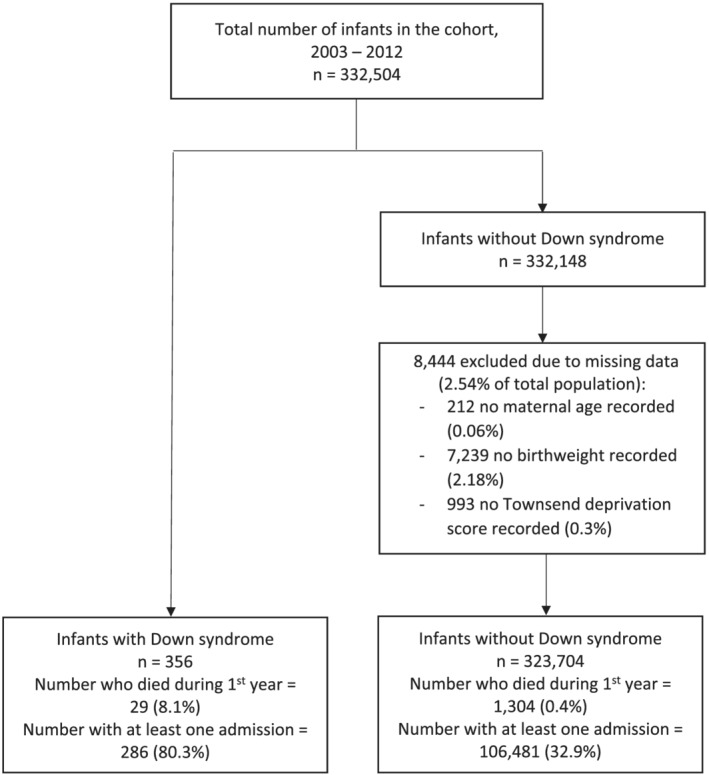
Cohort flow diagram

Characteristics of the mothers and infants including birth year, gender, UK Townsend Deprivation Score (grouped into quintiles, Yousaf and Bonsall 2017), maternal age (categorised in age ranges) and birthweight (classified as low, normal or high) were examined, stratified according to whether the infant had Down syndrome or not. Cases with any missing data on any of these covariates, or variables that had a large proportion of missing data, were excluded from the analysis (Fig. 1). To identify infants with other major congenital anomalies (i.e. anomalies that resulted in hospital admissions), we examined the first PEDW code for each admission in every child; if any of these included a code from the ‘Congenital malformations, deformations and chromosomal abnormalities’ ICD‐10 chapter (Q codes) that were not Down syndrome, we considered that the child had a major congenital anomaly.

### Statistical analysis

All inpatient admissions during the first year of life were included regardless of whether they were emergency, elective or other admissions. We first summarised the information on the first admission, and then examined all admissions in the first year of life. Secondary analyses include stratifying the data by type of admission (e.g. emergency) and timing of admission, that is, neonatal or post‐neonatal period. We also examined patterns in the primary reason for the hospital admissions, and the duration of these admissions. We then stratified the cohort for the presence or absence of Down syndrome and also the presence of other major congenital anomalies, resulting in four groups for analyses (Table 5).

We used Kaplan–Meier survival curves to examine the probability of at least one hospital admission in the first year of life, stratified according to whether the child had Down syndrome or not. Cox regression was used to compare time to first hospital admission between infants with and without Down syndrome, examining all first inpatient admissions in the first instance and then stratifying by the type of admission. Unadjusted hazard ratios, and those adjusted for gender, maternal age, birthweight and deprivation quintile, were examined. The proportional hazards assumption was assessed graphically using log‐minus‐log plots and was tested based on the Schoenfeld residuals. Hazard ratios were also adjusted for risk of multiple admissions, using Anderson–Gill models (White *et al*. 2011).

All analyses were conducted within the SAIL Gateway using Stata version 15.1.

## Results

### Incidence of Down syndrome

Figure 1 shows the cohort flow diagram. There were 332 504 infants in the cohort, of whom 356 were diagnosed with Down syndrome. There were 8444 (2.5%) excluded from the group without Down syndrome due to missing covariate data, leaving 324 060 in the final cohort for analysis. The characteristics and socio‐economic demographics are summarised in Table 2. The prevalence of Down syndrome between 2003 and 2011 was 1.1 per 1000 births. There were slightly more male than female participants in both the groups. Mothers of infants with Down syndrome were older, with 48.0% aged 35 or older at birth compared with only 16.1% of mothers of infants without Down syndrome. Of infants with Down syndrome, 27.0% had a low birthweight compared with 6.9% of infants without Down syndrome. The highest percentage of births overall was in mothers living in the most deprived quintile; however, there was a slightly higher proportion of infants with Down syndrome (20.5%) in the least deprived area category compared with infants without Down syndrome (16.2%). There were also almost twice the rate of multiple births and 10% more caesarean section births in infants with Down syndrome. The mortality rate in infants with Down syndrome was high at 8.1% (Fig. 1), more than 20 times higher than the general paediatric population (0.4%).

**TABLE 2:** Characteristics of the included cohort

Characteristic	Down syndrome	No Down syndrome
	Total: 356 (100%)	Total: 323 704 (100%)
Year of birth	*n* (%)	*n* (%)
2003–2007	165 (46.35)	158 700 (49.03)
2008–2012	191 (53.65)	165 004 (50.97)
Gender		
Male	188 (52.81)	166 053 (51.30)
Female	168 (47.19)	157 651 (48.70)
Maternal age (years)		
<25	72 (20.22)	100 908 (31.17)
25–29	51 (14.33)	88 406 (27.31)
30–34	62 (17.42)	82 133 (25.37)
35+	171 (48.03)	52 257 (16.14)
Birthweight (g)		
<2500 g (low)	96 (26.97)	22 360 (6.91)
≥2500 g (normal)	260 (73.03)	301 344 (93.09)
Deprivation quintile:		
1 (least deprived)	73 (20.51)	52 527 (16.23)
2	53 (14.89)	55 924 (17.28)
3	60 (16.85)	62 946 (19.45)
4	75 (21.07)	68 457 (21.15)
5 (most deprived)	95 (26.69)	83 850 (25.90)
Multiple births		
Yes (twins, etc.)	17 (4.78)	9330 (2.88)
No	339 (95.22)	314 374 (97.12)
C‐section		
Yes	119 (33.43)	78 740 (24.32)
No	237 (66.57)	244 964 (75.68)

### Incidence rate of admissions

**FIGURE 2:**
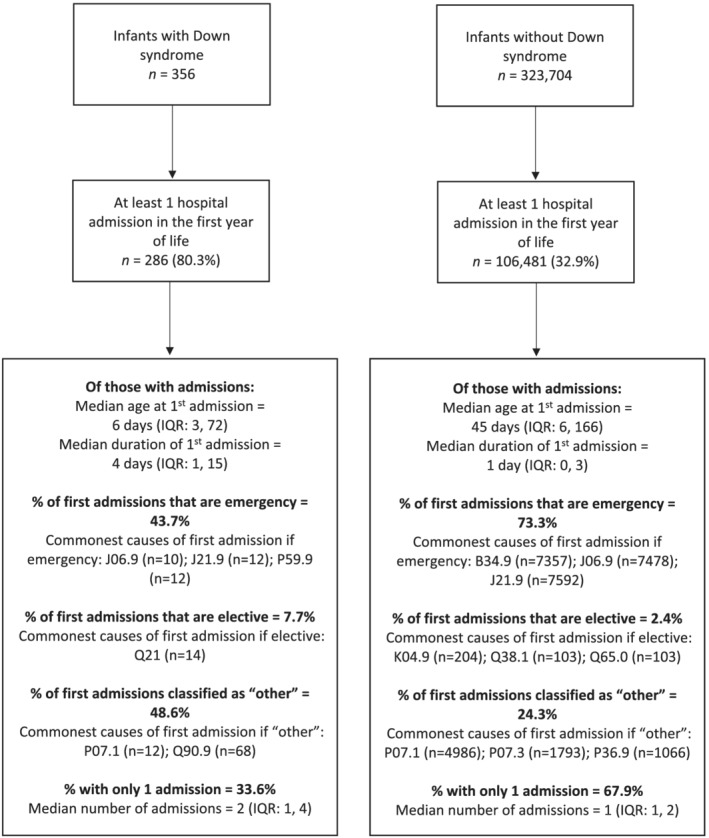
Admission characteristics in infants with and without Down syndrome

**FIGURE 3:**
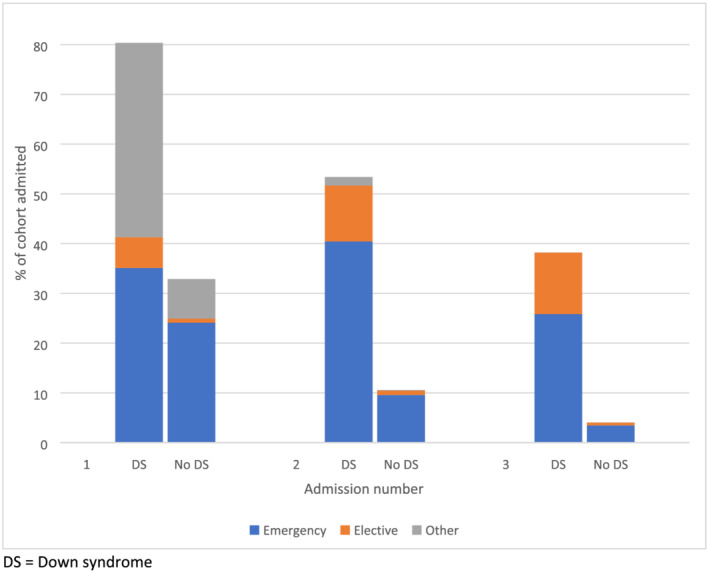
Proportion of infants with and without Down syndrome admitted, one to three admissions, by type of admission. DS, Down syndrome. [Colour figure can be viewed at wileyonlinelibrary.com]

Of the 356 infants with Down syndrome, 286 (80.3%) were admitted at least once during their first year of life, compared with 32.9% of infants without Down syndrome (Fig. 2). These first admissions were earlier [at a median of 6 days interquartile range (IQR) (3, 72) compared with 45 days (IQR 6, 166)] and longer [median of 4 days (IQR 1, 15) compared with 1 day (IQR 0, 3)] than in infants without Down syndrome. Of the first admissions in infants with Down syndrome, 43.7% were emergency admissions, 7.7% were elective and 48.6% were classified as ‘other’ (compared with 73.3%, 2.4% and 24.3% in infants without Down syndrome). Of those with at least one admission, 33.6% of infants with Down syndrome had only one admission in the first year of life [median number of admissions 1 (IQR 1, 4)], compared with 67.9% of infants without Down syndrome [median number of admissions 1 (IQR 1, 2)]. A higher proportion of infants with Down syndrome had 2nd and 3rd (and higher order) admissions, with a higher percentage being emergency admissions (Fig. 3).

In both groups, most first admissions occurred during the first month of life and admission rate decreased with age (Supporting information, Fig. S1; additional detail on the admissions in the neonatal and postnatal periods are given in Tables S1a and S1b). The risk of at least one hospital admission was almost four times greater in infants with Down syndrome compared with those without [adjusted hazard ratio (aHR) 3.77, 95% confidence intervals (CIs) 3.36–4.23] (Table 3). Accounting for multiple admissions using the Anderson–Gill models increased the risk to 5.65 (95% CI 5.00, 6.38) in infants with Down syndrome, as they were more likely to have multiple admissions (Table S2). The risk of at least one admission was greater in infants with Down syndrome regardless of the type of admission, with the risk of elective admissions being highest (aHR 16.70, 95% CI 10.95, 25.47).

**TABLE 3:** Hazard ratios for risk of at least one admission to hospital during the 1st year of life, in infants with and without Down syndrome

	Number with at least 1 admission	Unadjusted hazard ratio (95% CI)	Adjusted hazard ratio (95% CI)^†^
All first admissions			
Infants without Down syndrome	106 481	1.00	1.00
Infants with Down syndrome	286	4.60 (4.10, 5.17)	3.77 (3.36, 4.23)
If the first admission is an emergency			
Infants without Down syndrome	78 041	1.00	1.00
Infants with Down syndrome	125	3.34 (2.80, 3.98)	3.40 (2.85, 4.05)
If the first admission is elective			
Infants without Down syndrome	2587	1.00	1.00
Infants with Down syndrome	22	19.73 (12.97, 30.02)	16.70 (10.95, 25.47)
If the first admission is classified as ‘other’			
Infants without Down syndrome	25 828	1.00	1.00
Infants with Down syndrome	139	5.91 (5.00, 6.98)	2.93 (2.48, 3.46)

^†^ Adjusted for baby's gender, maternal age, Townsend deprivation quintiles and birthweight.

The proportional hazards assumption holds for each analysis.

CI, confidence interval.

### Cause of admissions

The most frequent causes of admission found in infants with Down syndrome in descending order were congenital malformations, respiratory diseases, conditions originating in the perinatal period and infectious diseases (Table 4). This was largely the same in infants without Down syndrome, although conditions originating in the perinatal period were the most common, followed by respiratory diseases. The most frequent causes of emergency admissions in both groups were of respiratory diseases, specifically an acute upper respiratory infection and acute bronchiolitis. Congenital anomalies were the commonest cause of elective admissions; however, it should be noted that most of these were coded as Down syndrome. With ‘other’ admission types, admissions originating in the perinatal period were prevalent in both cohorts.

**TABLE 4:** Commonest causes of admission in the first 12 months in infants with at least one admission per admission type

	Infants with Down syndrome			Infants without Down syndrome		
	Total number of infants^‡^ (%)	Total number of admissions (order^§^)	Median age at 1st admission	Total number of infants^‡^ (%)	Total number of admissions (order^§^)	Median age at 1st admission
a. All admissions						
All admissions	286 (100)	920^†^	6 (IQR 3, 72)	106 481 (100)	167 430^†^	45 (IQR 6, 166)
Congenital malformations (Q)	184 (64.3)	353 (1)		6926 (6.5)	10 308 (4)	
Respiratory diseases (J)	122 (42.6)	262 (2)		30 565 (28.7)	38 016 (2)	
Originating in the perinatal period (P)	89 (31.1)	98 (3)		36 145 (33.9)	39 289 (1)	
Certain infections (A + B)	56 (19.6)	72 (4)		22 626 (21.2)	26 042 (3)	
b. Emergency admissions						
All emergency admissions	222 (100)	592^¶^	65.5 (IQR 24, 154)	87 519 (100)	130 993^¶^	85 (IQR 30, 206)
Respiratory diseases (J)	121 (54.5)	258 (1)		30 369 (34.7)	37 669 (1)	
Congenital malformations (Q)	85 (38.3)	115^††^ (2)		2503 (2.9)	3150 (6)	
Certain infections (A + B)	56 (25.2)	72 (3)		22 454 (25.7)	25 794 (2)	
Originating in the perinatal period (P)	43 (19.4)	49 (4)		15 851 (18.1)	17 448 (3)	
c. Elective admissions						
All elective admissions	97 (100)	181^¶^	139 (IQR 105, 205)	6679 (100)	10 063^¶^	144 (IQR 84, 241)
Congenital malformations (Q)	83 (85.6)	149 (1)		3130 (46.9)	4754 (1)	
Gastro‐intestinal diseases (K)	7 (7.2)	9 (2)		1047 (15.7)	1144 (2)	
d. Other admissions						
All other admissions	140 (100)	147^¶^	3 (IQR 1, 4.5)	26 049 (100)	26 347^¶^	3 (IQR 1, 5)
Congenital malformations (Q)	85 (60.7)	89 (1)		2 387 (9.2)	2 401 (2)	
Originating in the perinatal period (P)	49 (35.0)	49 (2)		21 315 (81.8)	21 426 (1)	

^†^ Includes data from all admissions.

^‡^ Total number of infants with at least one admission of that type (the admission does not have to be the 1st admission, which is why the number is different to the number in previous tables of 1st admissions).

^§^ Position in terms of commonest causes for children with or without Down syndrome.

^¶^ Twenty‐one of 592 emergency admissions in children with Down syndrome have no cause information; 18 660 of 130 993 emergency admissions in children with Down syndrome have no cause information; <5 of 181 elective admissions in children with Down syndrome have no cause information; 688 of 10 063 elective admissions in children with Down syndrome have no cause information; 8 of 147 other admissions in children with Down syndrome have no cause information; 1457 of 26 347 other admissions in children with Down syndrome have no cause information.

^††^ Seventy‐one of these are coded as ‘Down syndrome’ but are included because they are coded as emergency admissions (we are uncertain of the underlying emergency).

### Effect of congenital anomalies

In the 286 infants with Down syndrome who had at least one hospital admission, a congenital anomaly Q code other than Down syndrome was noted to be the primary cause of an admission in at least one of their admissions for 94 (32.9%) infants, with all three of the commonest anomalies identifying a congenital heart condition (Table 5). A congenital anomaly was noted in the admission records of 6926 (6.5%) infants without Down syndrome, with the commonest anomalies being congenital hypertrophic pyloric stenosis, congenital non‐neoplastic naevus, and ventricular septal defects. Infants with Down syndrome had the earliest first admissions, regardless of whether they had a congenital anomaly [median first admission at 6 days (IQR 2, 72)] or not (median first admission at 6.5 days (IQR 3, 68)]. However, the burden of hospital admissions was highest overall in infants with Down syndrome and another congenital anomaly, with only 8.5% of this group having only one admission in the first year of life compared with 41.1% of infants with no Down syndrome and another congenital anomaly. Additionally, these children had a median of four admissions (IQR 3, 6), and a median total of 21 days in hospital (IQR 11, 47) in the first year of life compared with a median total of 4 days for infants with no Down syndrome and another congenital anomaly (IQR 2, 11). Infants with Down syndrome and no other congenital anomaly spent a median total of 7 days (IQR 2, 19) in hospital during their first year of life.

**TABLE 5:** Characteristics of admission according to the presence of congenital anomalies, in infants with at least one admission

	Infants with no Down syndrome and no congenital anomaly *n* = 99 555	Infants with Down syndrome and no congenital anomaly *n* = 192	Infants with no Down syndrome and a congenital anomaly^†^ *n* = 6926	Infants with Down syndrome and congenital anomaly^‡^ *n* = 94
Median age at 1st admission	48 days IQR = 6, 173	6.5 days IQR = 3, 68	12 days IQR = 3, 70	6 days IQR = 2, 72
% with only 1 admission	69.8%	45.8%	41.1%	8.5%
Median number of admissions	1, IQR = 1, 2	2, IQR = 1, 3	2, IQR = 1, 3	4, IQR = 3, 6
Median total number of days in hospital during 1st year of life	2, IQR = 1, 4	7, IQR = 2, 19	4, IQR = 2, 11	21, IQR = 11, 47

^†^ Three commonest anomalies = Q40.0 Congenital hypertrophic pyloric stenosis (609), Q82.5 Congenital non‐neoplastic naevus (536), Q21.0 Ventricular septal defect (434).

^‡^ Three commonest anomalies = Q21.2 Atrioventricular septal defect (80), Q21.0 Ventricular septal defect (36), Q21.1 Atrial septal defect (12).

IQR, interquartile range.

## Discussion

This study has quantified the burden of hospital admissions for a population‐based sample of infants with Down syndrome in Wales and compared this with infants without Down syndrome. The majority (80%) of infants with Down syndrome started their inpatient history within the first year of life, concurring with previous findings (Fitzgerald *et al*. 2013). They were also admitted earlier, more frequently and for longer periods than infants without Down syndrome. In both groups, most admissions occurred during the first month of life and admission rate decreased with age. The most common causes of admission in infants with Down syndrome were congenital abnormalities, infections (particularly of the respiratory system), and conditions originating in the perinatal period (such as neonatal jaundice), which is in agreement with previous studies (So *et al*. 2007; Fitzgerald *et al*. 2013; Zhu *et al*. 2013). The risk of elective admissions was particularly high in infants with Down syndrome, probably due to the high burden of congenital abnormalities found in this group. The presence of other congenital abnormalities increased hospitalisations in all infants, but more so in infants with Down syndrome who spent a median total of 21 days in hospital (IQR 11, 47) during their first year of life if they had a co‐occurring congenital anomaly identified from their hospital admission data.

### Strengths and limitations

This was a large population‐based study that was representative of the general population in Wales, made possible with data linkage. The prevalence rate of Down syndrome found in this study of 1.1 per 1000 births is concurrent with previous literature (Weijerman and Winter 2010). CARIS is a complete register of all cases of Down syndrome in Wales. The infant mortality rate of 8% that we found in infants with Down syndrome in this cohort was comparable with previous reports of a similar period (Kucik *et al*. 2013). The cohort design allowed for comparisons of the burden of hospital admissions between infants with and without Down syndrome, unlike previous studies that were based on case‐series and may have selected high‐risk children (e.g. those followed up by hospital specialists) only.

Admission data were based on PEDW codes, which are routinely available data not collected for research purposes. Up to 14 codes per consultant episode can be completed; however, we only had access to the first code, due to the way in which data were extracted for this analysis. This should indicate the primary reason for admission, although we know that this is not always the case. For example, Down syndrome was a common primary cause of admission in infants with Down syndrome. Although this is likely to be related with the cause of admission, it is unlikely to be the primary reason for a child presenting at hospital. There was also a notable proportion of admissions with no diagnosis code (12.4%) (refer to the table notes in Table 4), which means that there is some uncertainty about the patterns of causes presented in this paper.

We included 10 years of data, which makes this a large cohort overall. However, the number of infants with Down syndrome with at least one hospital admission (*n* = 286) was relatively small and was further reduced when the cohort was stratified according to the presence of congenital abnormalities. This made it difficult to conduct sub‐group analyses and limits the precision of the estimates from the analysis. Furthermore, the presence of congenital abnormalities other than Down syndrome was determined by whether an infant had a hospital admission with a PEDW code beginning with ‘Q’ (the ICD‐10 chapter heading for congenital malformations, deformations and chromosomal abnormalities), other than the codes for Down syndrome. Therefore, only congenital anomalies for which infants had an admission during the first year of life, and where this anomaly was noted as the primary cause of the admission, were identified using this method. We have not identified all children with congenital anomalies using this method, especially those which may have been associated with less severe health problems. Despite this, the differences in hospitalisation patterns between children with Down syndrome with or without congenital abnormalities are comparable with other studies (Frid *et al*. 2002; Zhu *et al*. 2013).

There are also other issues with using linked routine data. In accordance with SAIL guidelines, we did not have access to accurate dates of births (all births within a week were given the date of the Monday of that week). We acknowledge that this introduces some inaccuracy in our measurements, specifically when calculating the time to first admission and when stratifying the data into neonatal versus post‐neonatal periods, although this measurement bias is not differential between children with and without Down syndrome as the change is applied in the same way throughout the cohort.

We also acknowledge that children with intellectual disabilities may be more likely to be admitted to hospital than children without (Mahon and Kibirige 2004), which affects 1 in 10 children with Down syndrome (Bittles *et al*. 2002). Our study investigates admissions up to age 1 year where this may not yet be identifiable (Mahon and Kibirige 2004). For children with intellectual disabilities, parental perception and social circumstances may also play a part in the clinician's decision to admit. Issues with feeding may relate to intellectual disabilities or may be part of the complex set of conditions associated with Down syndrome.

### Implications for clinical care

Guidelines for healthcare supervision in children with Down syndrome (Bull 2011; Down Syndrome Medical Interest Group 2018) have been developed to support physicians in the management of this population. Recommendations include screening for several conditions such as cataracts, heart defects and hearing problems during early childhood. This analysis confirms that children with Down syndrome suffer from a wide range of conditions in infancy, with the presence of congenital anomalies being a significant factor of higher admission rates and duration. Health services need to be responsive to the complex needs of these children. More attention should be focused on the first month of life, which is a crucial time as a significantly high proportion of children are being admitted. Down syndrome has been identified as a risk factor for severe respiratory‐syncytial virus infection due to physiological abnormalities and an immature immune system (Bloemers *et al*. 2007), and an increased risk for severe infections could be a factor for their prolonged stay in hospital. The results of this study emphasise the need for more support for children with Down syndrome regarding the prevention and management of infections, especially respiratory tract infections. Lastly, medical complications in children with Down syndrome is one of the most important challenges perceived by parents (Hanson 2003; Rahimi and Khazir 2019); therefore, it is necessary for clinicians to provide up‐to‐date information when counselling parents. Parents are in need of better quality information provided by healthcare providers regarding a diagnosis of Down syndrome (Skotko *et al*. 2009a; Skotko *et al*. 2009b). Their role as carers is a significant factor in the quality of care, and this in turn is affected by their knowledge. It has also been recognised that the health of the parents or carers are linked to the health of the affected children (Minnes and Steiner 2009). Parents should be equipped with the necessary knowledge so that they can advocate on behalf of their children, successively improving the health and quality of life of their children and also their own.

### Implications for future research

More research is necessary to determine why infants with Down syndrome have longer admissions than children without Down syndrome for the same causes. Immunological abnormalities such as thymic atrophy and reduced lymphocytes found during the neonatal period (de Hingh *et al*. 2005; Guaraldi *et al*. 2017), and certain anatomical features including a flattened nasal bridge, large protruding tongue and small mouth, have been suggested to increase susceptibility to respiratory problems in children with Down syndrome (Lam *et al*. 2010). It is necessary to further understand the risk factors present in children with Down syndrome that predispose them to infections and certain conditions as these may help in understanding why they are experiencing not just more frequent, but also longer hospital admissions compared with children without Down syndrome. We were able to perform exploratory analyses on hospitalisation patterns between infants with and without Down syndrome in the presence of other congenital anomalies. Future research is needed with better data on congenital anomalies to examine whether outcomes in children with and without Down syndrome and the same additional congenital anomalies are different. Previous studies have shown that the mortality rate is higher in children with Down syndrome with congenital heart disease and more so when the condition is severe (Dawson *et al*. 2014). There is currently limited evidence on other conditions and the medical burden associated with these. This is key to understanding the factors that affect survival as well as medical burden in this population.

## Conclusion

This study provides new population‐based evidence on hospitalisations in infants with and without Down syndrome. Children with Down syndrome have an elevated risk of hospital admissions in infancy, with more frequent and longer admissions. Respiratory tract infections and congenital heart disease remain a major burden in this population. More research is needed to understand how to better identify and treat these conditions particularly in the first month of life when most admissions occur.

## Acknowlegements

This research was supported by The Centre for the Development and Evaluation of Complex Interventions for Public Health Improvement (DECIPHer), a UK Clinical Research Collaboration Public Health Research Centre of Excellence, and The Centre for the Improvement of Population Health through E‐records Research (CIPHER). CIPHER was funded by: Arthritis Research UK, the British Heart Foundation, Cancer Research UK, the Chief Scientist Office (Scottish Government Health Directorates), the Economic and Social Research Council, the Engineering and Physical Sciences Research Council, the Medical Research Council, the National Institute for Health Research, the National Institute for Social Care and Health Research (Welsh Government), and the Wellcome Trust (Grant reference: MR/K006525/1). The development of the Wales Electronic Cohort for Children (WECC) was supported by a Translational Health Research Platform Award from the National Institute for Social Care and Health Research (grant reference: TPR08‐006).

## Source of Funding

This research was supported by The Centre for the Development and Evaluation of Complex Interventions for Public Health Improvement (DECIPHer), a UK Clinical Research Collaboration Public Health Research Centre of Excellence, and The Centre for the Improvement of Population Health through E‐records Research (CIPHER). CIPHER was funded by: Arthritis Research UK, the British Heart Foundation, Cancer Research UK, the Chief Scientist Office (Scottish Government Health Directorates), the Economic and Social Research Council, the Engineering and Physical Sciences Research Council, the Medical Research Council, the National Institute for Health Research, the National Institute for Social Care and Health Research (Welsh Government), and the Wellcome Trust (Grant reference: MR/K006525/1). The development of the Wales Electronic Cohort for Children (WECC) was supported by a Translational Health Research Platform Award from the National Institute for Social Care and Health Research (grant reference: TPR08‐006).

## Conflict of interest

No conflicts of interest have been declared.

## Supporting information


**FIGURE S1.** Kaplan–Meier curve of time to first admission in the first year of life, in infants with and without Down syndrome

**TABLE S1.** Admission characteristics in the neonatal (Table 1a) versus the post‐neonatal period (Table 1b)

**TABLE S2.** Hazard ratios for risk of multiple admission to hospital during the 1^st^ year of life, in infants with and without Down syndrome (Anderson‐Gill models)

## Data Availability

The data used in this study are available in the Secure Anonymised Information Linkage (SAIL) databank at Swansea University, Swansea, UK. SAIL has established an application process to be followed by anyone who would like to access data via SAIL (https://www.saildatabank.com/application process). All proposals to use SAIL data are subject to review by an independent Information Governance Review Panel (IGRP). Before any data can be accessed, approval must be given by the IGRP. The IGRP gives careful consideration to each project to ensure proper and appropriate use of SAIL data. When access has been granted, it is gained through a privacy‐protecting safe haven and remote access system referred to as the SAIL Gateway. Relevant information to allow acquisition of a replicable dataset is available in the paper, or can be requested from the authors. Please contact SAILDatabank@swansea.ac.uk for more detail on data access requests.
