# Supplementary material for: Hospital admissions in infants with Down syndrome: a record‐linked population‐based cohort study in Wales
Source: J Intellect Disabil Res. 2021 Dec 3;66(3):225–39. doi: 10.1111/jir.12903 (PMC9376940; doi:10.1111/jir.12903)

**SUPPLEMENTARY APPENDIX FIGURE 1.** Kaplan-Meier curve of time to first admission in the first year of life, in infants with and without Down syndrome


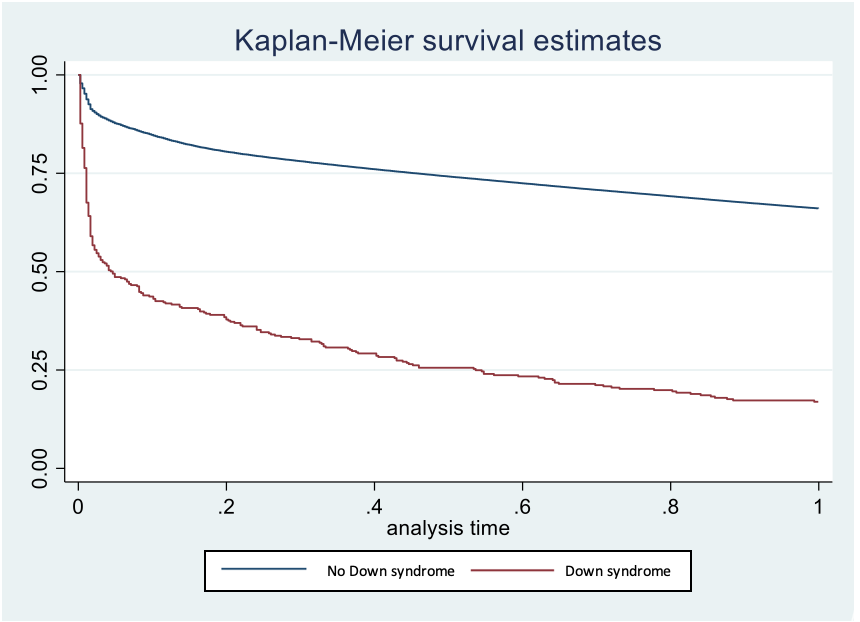

Supplement: Supplementary file 1 — FIGURE S1. Kaplan–Meier curve of time to first admission in the first year of life, in infants with and without Down syndrome [file JIR-66-225-s003.docx]
