# Supplementary material for: Hospital admissions in infants with Down syndrome: a record‐linked population‐based cohort study in Wales
Source: J Intellect Disabil Res. 2021 Dec 3;66(3):225–39. doi: 10.1111/jir.12903 (PMC9376940; doi:10.1111/jir.12903)
Supplement: Supplementary file 2 — TABLE S1. Admission characteristics in the neonatal (Table 1a) versus the post‐neonatal period (Table 1b) [file JIR-66-225-s002.docx]

**SUPPLEMENTARY APPENDIX TABLE 1.** Admission characteristics in the neonatal (table 1a) versus the post-neonatal period (table 1b, next page)

| **1a. Admissions in the neonatal period^*^** | | |
| --- | --- | --- |
|  | **Infants with Down syndrome**  **n = 356** | **Infants without Down syndrome**  **n = 323,704** |
| % with at least 1 admission in the neonatal period | 52.8% | 13.7% |
| Univariate hazard ratio for time to 1^st^ neonatal admission  (95% CI)^†^ | 5.19  (4.50, 5.99) | 1.00 |
| Multivariate hazard ratio for time to 1^st^ neonatal admission  (95% CI)^†^ | 3.26  (2.82, 3.76) | 1.00 |
| **Of those with neonatal admissions** | | |
| Total admissions that started in the neonatal period | 213 admissions in 188 neonates | 47,681 admissions in 44,415 neonates |
| % with only 1 admission in the neonatal period | 87.2% | 93.2% |
| Mean age at 1^st^ neonatal admission | 6.7  SD = 6.9 | 8,1  SD = 7.3 |
| Median age at 1^st^ neonatal admission | 4  (IQR 2, 9) | 5  (IQR 3, 12) |
| Mean number of admissions in the neonatal period | 1.1  SD = 0.4 | 1.1  SD = 0.3 |
| Median number of admissions in the neonatal period | 1  (IQR 1, 1) | 1  (IQR 1, 1) |
| Main causes of admissions in the neonatal period^‡^ | 1. Congenital anomalies (109)  2. Perinatal period codes (85) | 1. Perinatal period codes (35,409)  2. Congenital anomalies (3,237) |

* All admissions that started during the neonatal period.

^†^ Proportional hazards assumption holds.

^‡^ No cause noted for 11 of 213 admissions in children with Down syndrome that started in the neonatal period; no cause notes for 3,940 of 47,681 admissions in children without Down syndrome that started in the neonatal period; top 3 causes of neonatal admissions in infants with Down syndrome = Q90.0 (83), P59.9 (17), P07.1 (14); top 3 causes of neonatal admissions in infants without Down syndrome = P07 (7,735), P59.9 (4,782), P36.9 (1,281).

| **1b. Admissions in the post-neonatal period^**^** | | |
| --- | --- | --- |
|  | **Infants with Down syndrome**  **n = 341** | **Infants without Down syndrome**  **n = 320,191** |
| % with at least 1 admission in the post-neonatal period | 64.5% | 24.1% |
| Univariate hazard ratio for time to 1^st^ post-neonatal admission (95% CI)^††^ | 3.93  (3.45, 4.49) | 1.00 |
| Multivariate hazard ratio for time to 1^st^ post-neonatal admission (95% CI)^††^ | 3.81  (3.34, 4.35) | 1.00 |
| **Of those with post-neonatal admissions** | | |
| Total admissions that started in the post-neonatal period | 707 admissions in 220  infants | 119,749 admissions in 77,028 infants |
| % with only 1 admission in the post-neonatal period | 32.7% | 69.2% |
| Mean age at 1^st^ post-neonatal admission | 177.9  SD = 92.1 | 172.6  SD = 102.0 |
| Median age at 1^st^ post-neonatal admission | 172  (IQR 98, 253) | 162  (IQR 77, 261) |
| Mean number of admissions in the post-neonatal period | 3.2  SD = 2.8 | 1.5  SD = 1.3 |
| Median number of admissions in the post-neonatal period | 2  (IQR 1, 4) | 1  (IQR 1, 2) |
| Main causes of admissions in the post-neonatal period^‡‡^ | 1. Congenital anomalies (Q codes, 244)  2. Respiratory (J codes, 258)  3. Infectious diseases (A & B codes, 71)  4. Gastrointestinal disease (K codes, 47) | 1. Respiratory (J codes, 36,722)  2. Infectious diseases (A & B codes, 24,054)  3. Gastrointestinal disease (K codes, 12,545)  4. Congenital anomalies (Q codes, 7,071) |

** All admissions that started in the post-neonatal period; excludes 3,528 infants who died or were lost to follow-up during neonatal period

^††^ Proportional hazards assumption holds

^‡‡^ No cause noted for 19 of 707 admissions in children with Down Syndrome that started in the post-neonatal period; no cause notes for 16,873 of 119,749 admissions in children without Down Syndrome that started in the post-neonatal period; top 3 causes of post-neonatal admissions in infants with Down Syndrome = J21 (131), Q21 (109), Q90 (89); top 3 causes of post-neonatal admissions in infants without Down Syndrome = J21 (15,788), J06.9 (12,665), B34.9 (12,566)

**ICD 10 codes:**

B34.9 = viral infection, unspecified; J06.9 = acute upper respiratory tract infection, unspecified; J21 = acute bronchiolitis; P07 = disorders related to short gestation and low birthweight, not elsewhere classified; P07.1 = other low birthweight; P36.9 = bacterial sepsis of newborn, unspecified; P59.9 = neonatal jaundice, unspecified; Q21 = congenital malformations of cardiac septa; Q90 = Down syndrome.
