# Supplementary material for: Hospital admissions in infants with Down syndrome: a record‐linked population‐based cohort study in Wales
Source: J Intellect Disabil Res. 2021 Dec 3;66(3):225–39. doi: 10.1111/jir.12903 (PMC9376940; doi:10.1111/jir.12903)
Supplement: Supplementary file 3 — TABLE S2. Hazard ratios for risk of multiple admission to hospital during the 1st year of life, in infants with and without Down syndrome (Anderson‐Gill models) [file JIR-66-225-s001.docx]

**SUPPLEMENTARY APPENDIX TABLE 2.** Hazard ratios for risk of multiple admission to hospital during the 1^st^ year of life, in infants with and without Down syndrome (Anderson-Gill models)

|  | **Unadjusted hazard ratio (95% CI)** | **Adjusted hazard ratio (95% CI)^*^** |
| --- | --- | --- |
| **All admissions** | | |
| Infants without Down syndrome | 1.00 | 1.00 |
| Infants with Down syndrome | 7.26 (6.48, 8.13) | 5.65 (5.00, 6.38) |

* Adjusted for baby’s gender, maternal age, Townsend deprivation quintiles and birthweight.

The proportional hazards assumption holds for each analysis.
